# Pelvic ring reconstruction with a double-barreled free vascularized fibula graft after resection of malignant pelvic bone tumor

**DOI:** 10.1007/s00402-015-2197-7

**Published:** 2015-03-21

**Authors:** Koichi Ogura, Minoru Sakuraba, Shimpei Miyamoto, Tomohiro Fujiwara, Hirokazu Chuman, Akira Kawai

**Affiliations:** 1 Department of Musculoskeletal Oncology, National Cancer Center Hospital, 5-1-1 Tsukiji, Chuo-ku, Tokyo, 104-0045 Japan; 2 Department of Plastic and Reconstruction Surgery, National Cancer Center Hospital, 5-1-1 Tsukiji, Chuo-ku, Tokyo, 104-0045 Japan

**Keywords:** Internal hemipelvectomy, Pelvic ring reconstruction, Vascularized fibula graft, Complication

## Abstract

**Introduction:**

In patients undergoing limb-salvage internal hemipelvectomy, pelvic ring reconstruction is mandatory to maintain the stability of the pelvis and the spinal column, which finally expected to achieve a good functional outcome. However, no optimal reconstruction method has been established. In addition, no previous reports have highlighted the long-term complications of pelvic ring reconstruction after internal hemipelvectomy. We aimed to analyze the outcome of pelvic ring reconstruction using a double-barreled free vascularized fibula graft (VFG) after internal hemipelvectomy with special reference to long-term complications.

**Materials and methods:**

We conducted a retrospective review of 9 consecutive patients (5 male, 4 female; mean age 31 years) who underwent pelvic ring reconstruction using a double-barreled free VFG after internal hemipelvectomy (P1, *n* = 4; P1 + 4, *n* = 3; P1 + 2, *n* = 2) at our institution between 1998 and 2013. The mean follow-up period was 55 months (range 3–131 months).

**Results:**

The mean length of the bone defect was 9 cm. The methods of fixation included a Cotrel-Dubosset rod (*n* = 4), screw (*n* = 3), and screw and plate (*n* = 2). Bone union was achieved in 5 of 8 patients (63 %) over a 1-year follow-up. The mean period required for bone union was 5.4 months (range 3–7 months). There were 3 early postoperative complications: 2 deep infections resulting in graft removal and 1 implant failure resulting in non-union. Among 3 patients, 2 developed scoliosis within 5 years. One patient developed lumbar disc hernia as a result of scoliosis, for which surgical intervention was required. The mean Musculoskeletal Tumor Society score was 57 % at the last follow-up.

**Conclusions:**

In conclusion, this reconstruction method can achieve an early and high rate of bone union and provide good functional outcome. However, follow-up with careful attention to postoperative complications, including deep infection in the early postoperative period and spinal deformity in the long term, is necessary.

## Introduction

Limb-salvage surgery has replaced amputation for radical treatment of malignant pelvic tumors along with the introduction of effective chemotherapy, imaging modalities and modern surgical techniques. However, limb-salvage surgery for malignant pelvic tumors is still challenging because of their large size at diagnosis, the complex anatomy of the pelvis, and closely-situated vital organs such as vessels, nerve bundles, and viscera. In patients undergoing limb-salvage internal hemipelvectomy, pelvic reconstruction is mandatory to maintain the stability of the pelvis and the spinal column to reestablish a continuity of the ilium, the sacrum, and the pubis, which finally expected to achieve a good functional outcome.

Although several reconstruction methods including the use of vascularized iliac autografts [1, 2], strut allografts [1, 3], gluteus medius muscle pedicle iliac bone grafts [4], or vascularized or non-vascularized fibula grafts (VFG or NVFG) [5–7] have been reported for connecting the remaining periacetabular bone to the sacrum, no optimal reconstruction methods have been standardized. In addition, no previous reports have described the long-term outcomes of pelvic ring reconstruction after internal hemipelvectomy from the viewpoint of adverse events.

The aim of the present study was to analyze the clinical and functional outcomes of pelvic ring reconstruction using a double-barreled free VFG after internal hemipelvectomy with special reference to the long-term outcomes.

## Patients and methods

We conducted a retrospective review of 9 consecutive patients who underwent pelvic ring reconstruction using a double-barreled free VFG after internal hemipelvectomy at our institution between 1998 and 2013. Their clinical data and treatment outcomes were analyzed retrospectively.

The following data were examined: histologic diagnosis, surgical details (resection type, length of bone defect, and reconstruction details), adjuvant therapy (chemotherapy and radiotherapy), postoperative complications (e.g., non-union, fracture, implant failure, or infection), time required for bone union, oncologic outcome, and functional outcome.

The procedure was performed with the patient in a “sloppy” lateral position. After resection of the tumor, the length of the bony defect was measured. The plastic surgery team harvested the ipsilateral fibular graft and the fibula was osteotomized to provide two struts for the reconstruction. The segment of bone proximal to the vascular pedicle was placed in a more superficial position in the pelvis to facilitate microvascular anastomoses. This superficial portion was usually the longer of the two fibular struts. The vascular pedicle was anastomosed with the superior gluteal artery/vein, the lumbar artery/vein, or the deep inferior epigastric artery/vein by the plastic surgeons. Afterward, orthopaedic surgeons fixed each graft to the sacrum and periacetabular bone so that the end of the fibula was placed within an appropriately same sized burr hole in the remaining bone. The fixation of the transferred bone graft was performed using screws and a plate in early 3 cases (Case 1, 2, and 3). However, more recently, our first choice of fixation device has been the Cotrel-Dubousset rod (C-D rod) system, which was originally developed to treat spinal scoliosis. The system consists of a rod and pedicle screws and can be used for internal fixation of traumatic lesions of the spine and for pelvic ring reconstruction. The advantage of the device is that transferred bone can be rigidly fixed by compression pressure along the rod, resulting in early rehabilitation of walking. However, we did not use the device in Case 8 and 9 because it is relatively large for pediatric patients. When the C-D rod system was used for fixation between the stump of the sacrum and the ilium, compression pressure was added along the rod to fix rigidly.

We attempted wide resection of the primary tumor with a negative surgical margin whenever possible. The surgical margin was determined histologically at the point closest to the area resected, and was classified as negative (no residual disease) or positive (presence of microscopic residual disease at the inked margin). The types of resection were determined according to the classification of pelvic resections proposed by Enneking et al. [8]. Type I resection (P1) are those involving the ilium; type II resection (P2), those involving the acetabular bone; type III resection (P3), those involving the pubis and ischium; and type IV resection (P4), those involving the unilateral sacrum.

Bone union was assessed in 8 patients who could be followed up over 1 year and 1 patient who could not be sufficiently evaluated the bone union due to the short follow-up duration was excluded from the assessment (1 patient (Case 2) who died at 3 months postoperatively). Bone union was assessed using monthly plain radiographs and CT at 1- to 3-month interval.

Functional outcome of the reconstructed limb was assessed using the Musculoskeletal Tumor Society (MSTS) scoring system [9], which included pain, function, emotional acceptance, the use of any external support, walking ability, and gait alteration.

## Results

Patient demographics, adjuvant therapy, and oncologic outcomes are summarized in Table 1. There were 5 males and 4 females with a mean age of 31 years (range 10–52 years). The mean tumor size was 10 cm (range 5–14 cm). The histological diagnoses were chondrosarcoma (grade 1) (*n* = 4), Ewing’s sarcoma (*n* = 3), and osteosarcoma (*n* = 2). None of the patients had any preoperative comorbidities, such as diabetes mellitus, that may have predisposed them to poor bone union. The mean follow-up period was 55 months (range 3–131 months). Adjuvant radiotherapy and chemotherapy were performed in 2 and 5 patients, respectively. Oncological outcomes at the time of last follow-up were CDF in 5 patients, NED in 1, and DOD in 3.

**Table 1:** Patient demographics and adjuvant therapy data

No.	Age	Sex	Tumor size (cm)	Histologic diagnosis	Chemotherapy	Radiotherapy	Local recurrence	Metastasis	Oncologic outcome	Follow-up period (months)
1	43	M	11	Osteosarcoma	Preoperative/postoperative	None	Yes	Yes	DOD	23
2	14	M	5	Ewing’s sarcoma	Preoperative/postoperative	Preoperative	No	Yes	DOD	3
3	18	F	6	Metastatic osteosarcoma	Preoperative/postoperative	None	Yes	Yes	DOD	33
4	52	F	12	Chondrosarcoma (grade 1)	None	None	No	No	CDF	131
5	32	M	13	Chondrosarcoma (grade 1)	None	None	No	No	CDF	126
6	44	M	14	Chondrosarcoma (grade 1)	None	None	No	No	CDF	96
7	36	M	12	Chondrosarcoma (grade 1)	None	None	No	No	CDF	63
8	14	F	10	Ewing’s sarcoma	Preoperative/postoperative	None	No	Yes	NED	16
9	10	F	7	Ewing’s sarcoma	Preoperative/postoperative	Postoperative	No	No	CDF	7

*DOD* dead of disease, *CDF* continuously disease-free, *NED* no evidence of disease

Surgical details are summarized in Table 2. All the patients underwent limb-salvage internal hemipelvectomy and subsequent pelvic ring reconstruction with a double-barreled free VFG. The types of resection included P1 (*n* = 4), P1 + 4 (*n* = 3), and P1 + 2 (*n* = 2). Negative surgical margins were achieved in 7 patients (78 %). The mean length of the bone defect was 9 cm (range 6–11 cm). The method of fixation included C-D rod system (*n* = 4), screw (*n* = 3), and screw and plate (*n* = 2). Soft tissue reconstruction was added in 3 patients. The total operation time ranged from 510 to 934 min (mean 736 min) and mean blood loss was 1834 ml (range 647–2754 ml). No significant intraoperative complications developed.

**Table 2:** Surgical details of the patients

No.	Type of resection	Surgical margin	Length of bone defect (cm)	Site of fixation (proximal/distal)	Method of fixation	Soft tissue reconstruction	Operation time (min)	Blood loss (ml)
1	P1 + 2	Negative	8	Sacrum/femoral head	Plate, screw		832	1900
2	P1 + 2	Positive	8	Sacrum/femoral head	Screw		635	2563
3	P1 + 4	Negative	11	Sacrum/periacetabular bone	Plate, screw		510	720
4	P1	Negative	9	Transverse process (L5), sacrum/periacetabular bone	C-D rod		890	2135
5	P1	Negative	11	Transverse process (L4, L5)/periacetabular bone	C-D rod	VRAM flap	715	2408
6	P1	Negative	9	Sacrum/periacetabular bone	C-D rod		882	2754
7	P1	Negative	8	Transverse process (L5), sacrum/periacetabular bone	C-D rod	VRAM flap	623	1309
8	P1 + 4	Negative	6	Sacrum/periacetabular bone	Screw	Free LD flap	934	2070
9	P1 + 4	Positive	9	Sacrum/periacetabular bone	Screw		604	647

*C-D rod* Cotrel-Dubosset rod, *VRAM flap* vertical rectus abdominis myocutaneous flap, *Free LD flap* Free latissimus dorsi flap

Treatment and functional outcomes are summarized in Table 3. Complete bone union was achieved in 5 of 8 patients (63 %) who could be followed up for over 1 year. The mean period required for bone union was 5.4 months (range 3–7 months). Three early postoperative complications occurred in 3 patients: 2 deep infections resulting in graft removal, and 1 implant failure and subsequent graft displacement resulting in non-union. Among 3 patients who had been followed up for over 5 years without graft removal (Case 4, 5, 7), 2 developed scoliosis (Case 4, 5). One patient developed lumbar disc hernia as a result of scoliosis, for which surgical intervention was required. The mean Musculoskeletal Tumor Society score was 57 % (range 23–87 %).

**Table 3:** Treatment, oncologic, and functional outcomes

No.	Time to bone union (months)	Postoperative complications		Additional surgery for complications	MSTS score (%)	Weight-bearing at last follow-up	Walking ability
		Early	Late				
1	3	None	NA (short follow-up)		23	FWB	Without cane
2	NA (DOD at 3 months postoperatively)	None	NA (short follow-up)		NA	Died before attempted	Died before attempted
3	NA (graft removal)	Infection	NA (graft removal)	Graft removal for infection, amputation for local recurrence	NA	FWB	Double crutches
4	5	None	Scoliosis		43	FWB	Without cane
5	7	None	Scoliosis resulting in lumbar disc hernia (L4/5)	Discectomy for lumbar disc hernia	53	FWB	Without cane
6	NA (graft removal)	Infection	NA (graft removal)	Graft removal for infection	83	FWB	Single cane
7	5	None	None		87	FWB	Without cane
8	Nonunion	Screw breakage	NA (short follow-up)		43	FWB	Without cane
9	7	None	NA (short follow-up)		27	FWB	Double crutches

*DOD* dead of disease, *NA* not available, *FWB* full-weight bearing

### Representative case (Case 5)

**Fig. 1:**
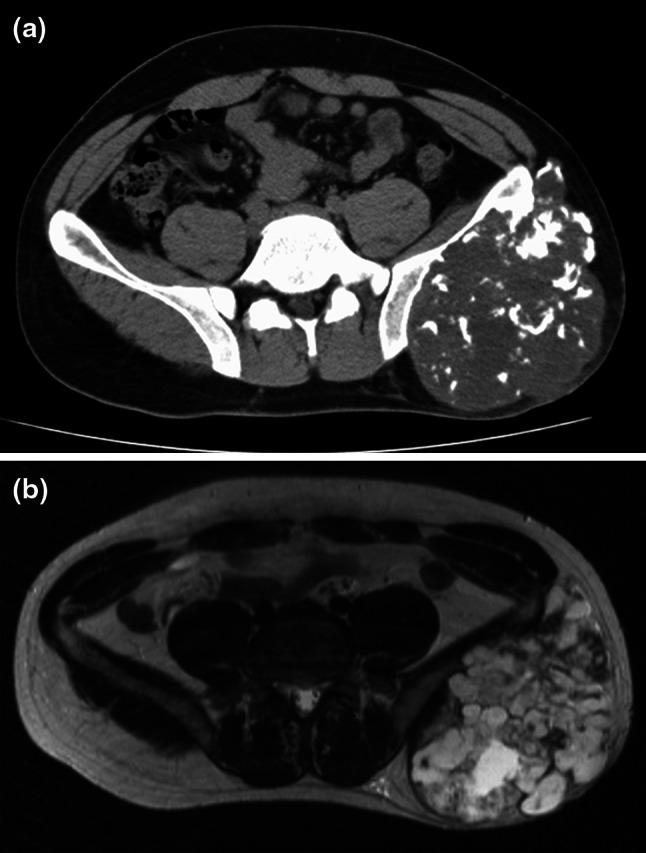
Axial CT shows an expansile tumor that originates from the posterior aspect of the ilium and contains foci of calcification (**a**). T2-weighted axial MR images demonstrate a lobulated lesion of the left ilium with heterogeneous high signal intensity, consistent with chondroid matrix (**b**)

**Fig. 2:**
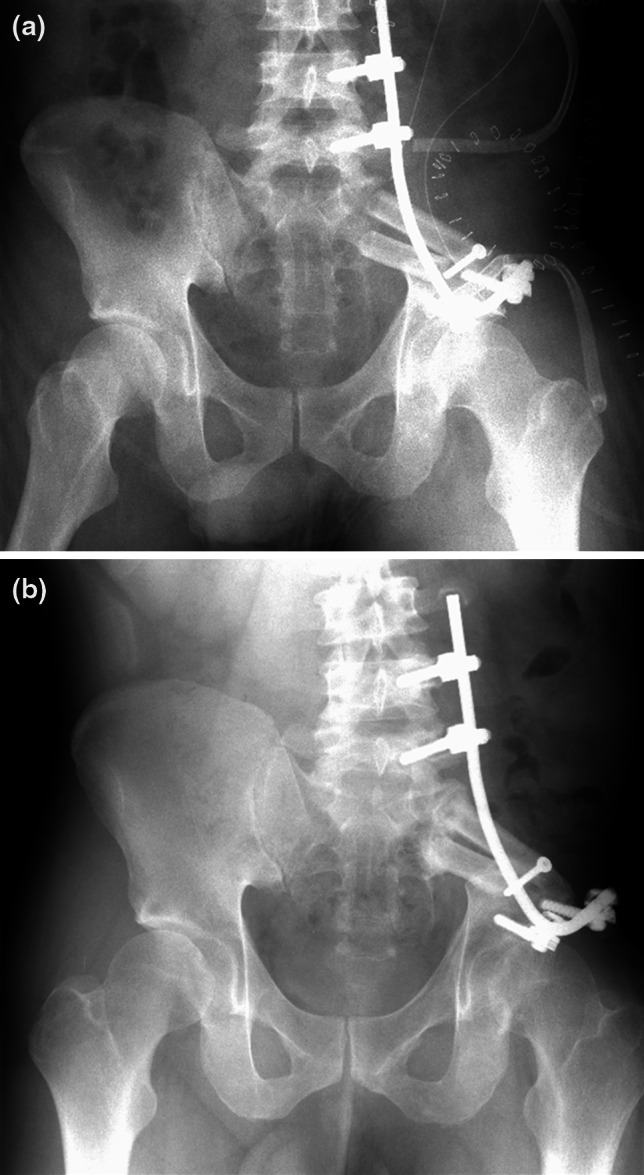
Internal hemipelvectomy (P1) and subsequent pelvic ring reconstruction with a double-barrel free vascularized fibular graft stabilized by a Cotrel-Dubosset rod is performed (**a**). Complete bone union was achieved at 7 months after surgery (**b**)

**Fig. 3:**
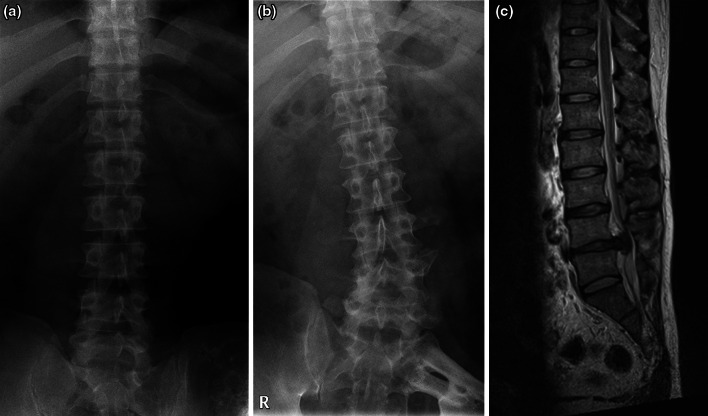
Plain radiograph at diagnosis of pelvic chondrosarcoma (**a**) and at diagnosis of lumbar disc hernia 6 years postoperatively (**b**). Gradual progression of scoliosis is evident. T2-weighted sagittal MR images demonstrate a severe lumbar disc hernia at the level of L4/5 (**c**)

The patient was a 32-year-old male with chondrosarcoma of the left ilium located mainly at the posterior side (Fig. 1a, b). An extraosseous bulging mass caused marked thinning of the skin. He underwent a P1 resection and subsequent reconstruction of the bone defect, measuring 11 cm in length, with a double-barreled VFG rigidly fixed with a C-D rod (Fig. 2a). Also soft tissue reconstruction using a vertical rectus abdominis myocutaneous flap was performed to provide better filling of the dead space created by tumor resection and to cover the exposed bone and prosthetic hardware with sufficiently well vascularized soft tissue [10]. The postoperative course was uneventful and the patient began to move in a wheelchair in 2 weeks and to walk with double crutches in 3 weeks after surgery. Complete bone union was achieved in 7 months after surgery (Fig. 2b). However, 6 years after surgery, he complained of lower back and left leg pain with severe numbness, and radiographic evaluation revealed gradual progression of scoliosis (Fig. 3a, b) and L4/5 lumbar disc hernia (Fig. 3c). After discectomy, the symptoms disappeared and the patient became ambulatory immediately. At the last follow-up, he was able to walk without a walking aid with full-weight bearing (MSTS score, 53 %).

## Discussion

Since the 1980s, limb-salvage internal hemipelvectomy has replaced amputation for radical treatment of malignant pelvic tumors along with the introduction of effective chemotherapy, imaging modalities and modern surgical techniques [11, 12]. A type 1 resection usually creates a bony pelvic ring defect leading to pelvic instability. Since resection without pelvic ring reconstruction has not been shown to be effective because of distortion and instability of the pelvic ring, likely resulting in poor long-term function [3], several reconstruction options to maintain the stability of the pelvis and allow ambulation have been reported [1–7].

Reconstructive options for the pelvic ring reported to date have included allografts [3, 13–15], autoclaved autografts [16], vascularized iliac autografts [2, 4], NVFG [5], or VFGs [6, 7]. Although reconstruction using allografts has been one of the most popular options, it was reported to be associated with a high rate of complications including fracture, non-union, and infection [3, 14, 15]. Beadel et al. [3] reported 4 cases of pelvic ring reconstruction using a fibula allograft, and only 2 patients (50 %) had long-lasting bone union. Nishida et al. [2] first reported 5 cases of pelvic ring reconstruction using a vascularized iliac autograft fixed with a pedicle screw and rod. In their series, bone union was achieved in all patients within a mean period of 5.4 months. Postoperative complication was seen in 1 patient (20 %), who developed skin necrosis resulting from pedicle screw protrusion. Recently, Nassif et al. [4] reported a similar technique in 6 patients, all of whom achieved bone union and a good functional outcome with a mean MSTS score of 72 %, although postoperative complications occurred in 4 patients (67 %): scar necrosis (*n* = 2), infection (*n* = 1), and screw breakage (*n* = 1). From these results, vascularized iliac autograft transfer seems to be a good reconstructive option for patients with a relatively small pelvic ring defect who have adequate remaining iliac bone to transfer. However, this technique cannot be used for patients requiring extensive bone resection with little remaining iliac bone to transfer.

Several investigators have reported good clinical and functional outcomes and fewer complications of pelvic ring reconstruction using a fibula autograft [5–7]. Chang et al. [6] reported 6 cases of pelvic ring reconstruction after internal hemipelvectomy defects had been reconstructed with a double-barreled VFG. Radiologic evidence of complete bone union was noted after a mean postoperative period of 8 months (range 5–18 months). One patient (17 %) developed screw breakage at the sacral junction, and no other postoperative complications including flap failure, fracture, or infection were noted. Five patients (83 %) resumed full ambulation without any assistance in 5–18 months. Akiyama et al. [5] reported 10 cases of pelvic ring reconstruction after internal hemipelvectomy defects that were reconstructed with single (*n* = 4) or double-barreled (*n* = 6) non-VFG. Although the upper graft did not unite in 4 of 6 cases with a double-barreled graft, the lower or single graft united in all cases after a mean postoperative period of 7.3 months (range 3–12 months). One patient (10 %) developed plate breakage, but no other postoperative complications including fracture or infection were noted. They reported good functional outcome (mean MSTS score of 75.4 %), and 6 patients (60 %) were able to walk without a cane, three needed a cane, and one was wheelchair-bound because of ongoing pelvic pain.

In our series, bone union rate was good (63 %) and it resulted in early ambulation and good functional outcome. In addition, it allows reconstruction of extensive bone defects in the iliosacral region with little remaining iliac bone, as a VFG can be harvested with a maximum length of 28 cm [6]. However, follow-up with careful attention to postoperative complications, including deep infection in the early postoperative period and spinal deformity in the long term, is necessary.

Despite the above-mentioned early postoperative good outcomes of pelvic ring reconstruction using a fibula graft, no previous reports have focused on the long-term outcomes, especially long-term complications. This may have resulted from the difficulty of long-term follow-up in patients undergoing malignant pelvic tumor surgery due to the rarity of the surgery, the high incidence of postoperative complications resulting in amputation or reconstruction breakage or removal, or relatively early death from exacerbation of the disease. In our series, among 3 patients who had been followed up for over 5 years, 2 developed scoliosis. One patient developed lumbar disc hernia as a result of scoliosis, for which surgical intervention was required. Although this reconstruction method can achieve an early and high rate of bone union and provide good functional outcome in the early postoperative period, follow-up with careful attention to the adjacent joint disorder, such as spinal deformity, is necessary in the long term, and surgical intervention should be considered as appropriate.

Certain limitations of this study should be noted. It had a retrospective design and was based on a small number of patients. However, it should be noted that the situations for which this procedure is warranted are limited, and thus the number of patients will always be small, as has been the case in previous studies [1–7]. The procedure usually takes a long time and the associated increased blood loss requires extensive transfusion, especially in patients who have bone marrow suppression due to preoperative chemotherapy. Moreover, when no soft tissue reconstruction is added, significant aesthetic deformity due to the different shape of the fibula graft from the iliac crest is seen [6]. Nevertheless, we believe that our results are valuable and warrant debate, since this is one of the largest series reporting the outcome of pelvic ring reconstruction using a fibula graft after pelvic tumor resection and the first report to describe the long-term complications of this procedure.

In conclusion, this reconstruction method can achieve an early and high rate of bone union and provide good functional outcome. However, follow-up with careful attention to postoperative complications, including deep infection in the early postoperative period and spinal deformity in the long term, is necessary and surgical intervention should be considered as appropriate to resolve these problems.
